# Biological stability of DNA methylation measurements over varying intervals of time and in the presence of acute stress

**DOI:** 10.1080/15592294.2023.2230686

**Published:** 2023-07-02

**Authors:** Abner T. Apsley, Qiaofeng Ye, Laura Etzel, Sarah Wolf, Waylon J. Hastings, Brooke C. Mattern, Sue Rutherford Siegel, Idan Shalev

**Affiliations:** a Department of Biobehavioral Health, The Pennsylvania State University, University Park, PA, USA; b Department of Molecular, Cellular and Integrative Biological Sciences, The Pennsylvania State University, University Park, PA, USA; c Department of Psychiatry, Tulane University School of Medicine, New Orleans, LA, USA

**Keywords:** DNA methylation, stability, temporal dynamics, acute psychosocial stress, early-life adversity, epigenetic clocks, immune cell estimators

## Abstract

Identifying factors that influence the stability of DNA methylation measurements across biological replicates is of critical importance in basic and clinical research. Using a within-person between-group experimental design (*n* = 31, number of observations = 192), we report the stability of biological replicates over a variety of unique temporal scenarios, both in the absence and presence of acute psychosocial stress, and between individuals who have experienced early life adversity (ELA) and non-exposed individuals. We found that varying time intervals, acute stress, and ELA exposure influenced the stability of repeated DNA methylation measurements. In the absence of acute stress, probes were less stable as time passed; however, stress exerted a stabilizing influence on probes over longer time intervals. Compared to non-exposed individuals, ELA-exposed individuals had significantly lower probe stability immediately following acute stress. Additionally, we found that across all scenarios, probes used in most epigenetic-based algorithms for estimating epigenetic age or immune cell proportions had average or below-average stability, except for the Principal Component and DunedinPACE epigenetic ageing clocks, which were enriched for more stable probes. Finally, using highly stable probes in the absence of stress, we identified multiple probes that were hypomethylated in the presence of acute stress, regardless of ELA status. Two hypomethylated probes are located near the transcription start site of the glutathione-disulfide reductase gene (*GSR*), which has previously been shown to be an integral part of the stress response to environmental toxins. We discuss implications for future studies concerning the reliability and reproducibility of DNA methylation measurements.

**Abbreviations:** DNAm – DNA methylation, CpG − 5’-cytosine-phosphate-guanine-3,’ ICC – Interclass correlation coefficient, ELA – Early-life adversity, PBMCs – Peripheral blood mononuclear cells, mQTL – Methylation quantitative trait loci, TSS – Transcription start site, GSR – Glutathione-disulfide reductase gene, TSST – Trier social stress test, PC – Principal component.

## 1. Introduction

Following the accelerated progress of biotechnologies that enable the quantification of DNA methylation (DNAm), the last few decades have witnessed an ever-growing increase in the number of published studies associating biological phenomena and diseases with aberrant DNAm [1–4]. Technologies such as the Illumina 450K and EPIC BeadChip arrays use oligonucleotide probes to quantify the methylation status of cytosine molecules from specific 5’-cytosine-phosphate-guanine-3’ (CpG) sites across the human genome. Given the growing use of DNAm in research, the technical reliability and validity of DNAm CpG probe measurements are of critical importance for various clinical and genomic applications.

Probe validities are often measured by comparing array values to whole-genome bisulphite sequencing methods (for more information about whole-genome bisulfite sequencing, see reference [5]), whereas probe technical reliability is often quantified using either the Pearson correlation coefficient or the interclass correlation coefficient (ICC) of technical replicates. The consequences of using technically unreliable probes in genomic analyses can be profound [6,7]. Studies that tested the reliability of DNAm probes have used repeated measurements from the same biological samples, thus assessing the *technical* reliability [6,8–11]. However, limited work has been done on assessing the *biological* reliability – or what we will hereafter refer to as the ‘stability’ – of repeated DNAm measurements across time in epigenetic studies [10].

Studying the stability of biological replicates of DNAm probe measurements is important because of the temporal and dynamic nature of DNAm. Probe measurements of brain tissue have been shown to oscillate both seasonally and diurnally [12,13]. DNAm has also been observed to change across other cyclical processes, such as with endometrial tissue across a typical female menstrual cycle [14]. Additionally, the stability of whole blood DNAm probes can be temporally unstable due to the combination of different methylation profiles of leukocyte subtypes [15,16] and diurnal fluctuations in leukocyte proportions [17,18]. The presence of temporal and cyclical variations in DNAm probe measurements demonstrates the need to assess probe stability at varying timescales.

In addition to temporal alterations, both global and local DNAm changes have been observed in response to acute stress [19,20]. Physiological stress responses are also modulated within individuals that have experienced early-life adversity (ELA) [21], including differences in gene expression [22]. Variability in the stress responses of ELA versus non-ELA individuals may be in part due to differential DNAm stability in the presence of acute stress. The stability of DNAm probes in the presence of acute stress (with or without ELA) has implications for research study designs in which individuals are subjected to physically or psychologically adverse environmental stimuli. Additionally, both the temporal variability and sensitivity of DNAm measurements to acute stress can affect the robustness of common algorithms/estimators that use DNAm information, such as epigenetic ageing clocks [23–27] or immune cell proportion estimators [28,29].

**Figure 1.**
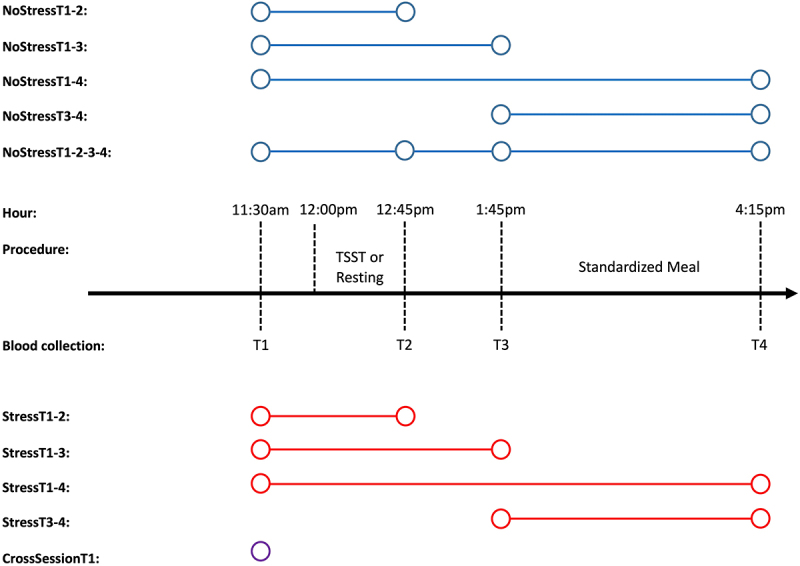
Description of test-retest scenarios.

Note: Blue lines and dots indicate the NoStress session and red lines and dots indicate the Stress session. CrossSessionT1 was the only scenario in which probes across sessions were used, which is represented by a purple open dot. Each open dot represents a time point that was used to calculate the probe stabilities under the corresponding scenarios. All scenarios included two time points to calculate probe stabilities, apart from NoStressT1-2-3-4, in which four time points were used.

Our study aimed to expand previous DNAm reliability research by reporting peripheral blood mononuclear cell (PBMC) probe stability over a variety of different temporal scenarios (see Figure 1 and **Table S1**) using the Infinium MethylationEPIC array. Scenarios were designed to test the stability of DNAm probes over varying lengths of time in both the absence of stress (NoStressT1–2: 75 minutes; NoStressT1–3: 135 minutes; NoStressT3–4: 150 minutes; NoStressT1–4: 285 minutes) and presence of acute psychological stress (StressT1–2: 75 minutes; StressT1–3: 135 minutes; StressT3–4: 150 minutes; NoStressT1–4: 285 minutes). Additionally, we tested the biological stability of probes measured 1 week apart (CrossSessionT1: 1-week baseline comparisons). First, we present how study design variables, such as sample size and number of repeated measures, relate to DNAm probe stability calculations. Next, we report how probe stability varied based on the temporal separation of biological measurements, as well as the presence of acute psychosocial stress. Comparisons of the stabilities of probes used in common epigenetic estimators/algorithms, which are biologically relevant, are then detailed in reference to the probe distributions for each temporal scenario. Finally, we present the specific effects of acute psychosocial stress on ‘highly stable’ probes (see Methods – Analysis Plan) and the effects of ELA on DNAm probe stability in the presence of acute stress.

## 2. Results

### 2.1. Probe stabilities varied based on type of ICC value, controlling for immune cell proportions, sample size, and number of repeated measures

**Figure 2.**
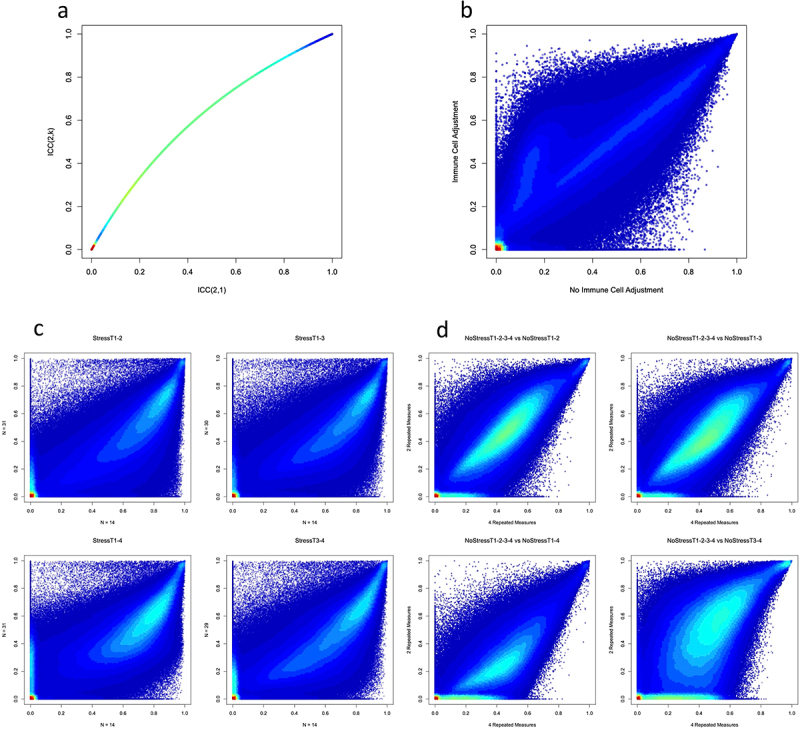
Associations of DNAm probe stability with sample characteristics.

Note: Heat-scatter plots are set on a three-colour gradient, with red being the densest, green being moderately dense, and blue being the least dense. White indicates no points in the region. A) Differences in probe stability associated with the method of calculating probe ICC values. B) Differences in probe stability associated with either adjusting or not adjusting the ICC values based on the proportions of immune cell subtypes. C) Four panels showing differences in probe stability associated with varying sample sizes (x-axis ICC with *N* = 14 samples; y-axis: ICC with *N* = 31 samples). D) Four panels show differences in probe stability associated with varying numbers of repeated measurements.

As there are multiple ways to measure the stability of DNAm probes [6,8], we compared two methods of calculating ICC values to determine which one to use as our stability measurement. In the NoStressT1–2 scenario (see Figure 1), stabilities calculated using the ICC(2,1) method showed a significantly lower value than those calculated using the ICC(2,k) method (*β*=-0.118, *P* < 0.001; Figure 2a). All other scenarios showed similar differences in stability (*β*=-0.129 ± 0.047) based on types of ICC values. To be conservative in our stability estimates, and because we used individual probe measurements instead of the mean of multiple measurements, all further analyses were conducted using ICC values calculated with the ICC(2,1) method (see [30] for a more detailed reasoning behind the choice of ICC type).

Using the ICC(2,1) method, we compared the stabilities computed with and without controlling for immune cell proportions in NoStressT1–2. Controlling for variations in immune cell proportions significantly increased the probe ICC values (*β* = 0.058, *P* < 0.001; Figure 2b). All other scenarios showed a variety of differences in stability (*β*_*range*_=[−0.042 to 0.053]) based on controlling for immune cell proportions. Probes with lower average stability were more sensitive to the effects of controlling for immune cell proportions than probes with higher stability. All further analyses were conducted using the ICC values adjusted for the proportion of immune cells.

Next, we tested whether the sample size was associated with probe ICC values. To do this, we compared the stability value distributions of groups of 14 randomly sampled individuals with a full sample of individuals from the same Stress scenarios. Stress scenarios were used for this analysis because the sample sizes were larger (*n* = 29–31) than the NoStress scenarios (*n* = 13–14). Contrary to expectations, in every scenario, larger sample sizes generally had lower probe ICC values (StressT1–2: *β*= −0.143, *P* < 0.001; StressT1–3: *β*= −0.120, *P* < 0.001; StressT1–4: *β*= −0.101, *P* < 0.001; StressT3–4: *β*= −0.120, *P* < 0.001; Figure 2c). These results were not driven by specific individuals because each ICC value was calculated using a different random sample of 14 individuals from the Stress scenario. Conversely, all scenarios with smaller sample sizes, when compared with larger sample sizes, had a greater number of probes with an ICC value less than 0.01 (*P* < 0.001). These results indicated that smaller sample sizes had more stable probes on average, but also a greater number of probes with very low stability. All further analyses were performed with randomly sampled smaller sample sizes for all Stress scenarios, to enable accurate comparisons between Stress and NoStress scenarios.

Finally, we compared the ICC values calculated using four repeated measures to the ICC values calculated using two repeated measures. To do so, we compared NoStressT1-2-3-4 with all other NoStress scenarios. Using more repeated measures significantly decreased probe ICC values, but with a small effect size for comparisons between NoStressT1-2-3-4 and NoStressT1–2 (*β*=-0.010, *P* < 0.001), NoStressT1–3 (*β*=-4.94E–04, *P* < 0.001), and NoStressT3–4 (*β*=-0.012, *P* < 0.001; see Figure 2d). However, when comparing NoStressT1-2-3-4 and NoStressT1–4, the latter of which included the same timeframe gap between measurements but with two repeated measures, we found that using four repeated measures significantly increased ICC values (*β* = 0.128, *P* < 0.001; see Figure 2d). Interestingly, when comparing Stress scenario ICC values calculated with four versus two repeated measures, we found that in all comparisons using four repeated measures significantly increased ICC values (StressT1–2: *β* = 0.151, *P* < 0.001; StressT1–3: *β* = 0.145, *P* < 0.001; StressT1–4: *β* = 0.151, *P* < 0.001; StressT3–4: *β* = 0.162, *P* < 0.001). In all comparisons, across both the NoStress and Stress scenarios, having more repeated measures corresponded to a significantly lower number of probes with ICC values less than 0.01 (*P* < 0.001).

### 2.2. Each test-retest scenario had a unique distribution of probe ICC values

**Figure 3.**
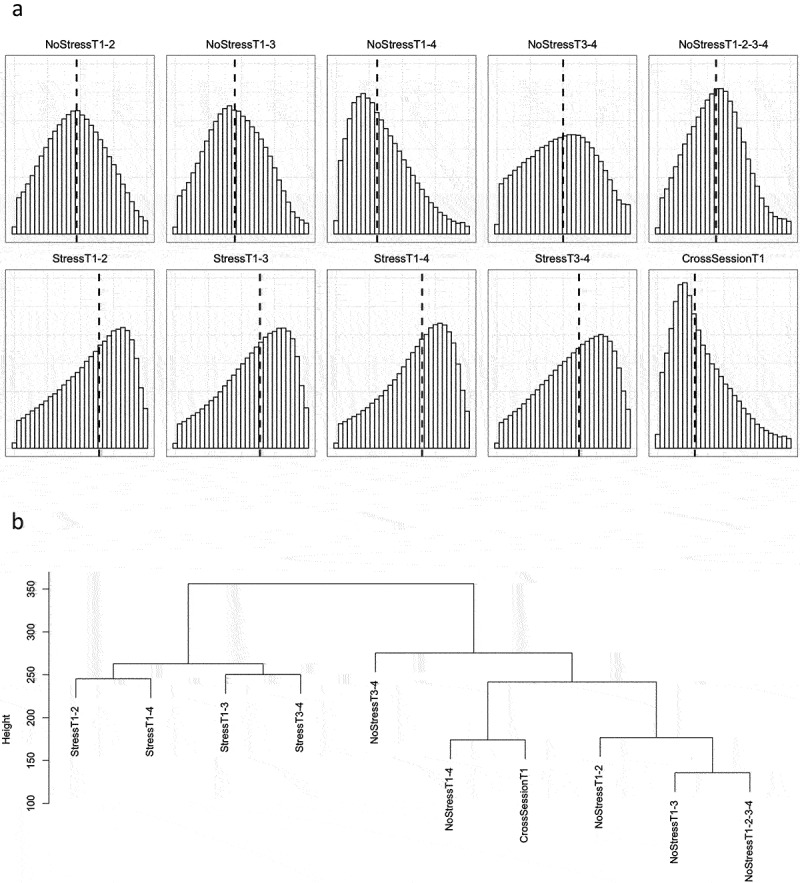
Stability of DNAm probes in test-retest scenarios.

Note: All probes with ICC values less than 0.01 were excluded from analyses in this figure. a) Distribution of probe stability for each test-retest scenario. The vertical dashed line indicates the median probe stability (excluding probes with ICC < 0.01). b) Cluster dendrogram showing the similarity of test-retest scenarios (based on probe ICC values).

DNAm probe stabilities were calculated for each of the ten previously mentioned scenarios (see Methods and Figure 1), and the distributions of ICC values for each scenario (excluding probes with ICC values less than 0.01) are shown in Figure 3a. Descriptive statistics for the probe stabilities of each scenario are shown in Table 1. Excluding probes with ICC < 0.01, the average median probe stability across scenarios was 0.50, with StressT1–4 having the highest median stability of 0.65, and CrossSessionT1 measurements over one week having the lowest stability of 0.28. To test whether probes with low ICC values had low ICC values across all scenarios, we computed Pearson correlation coefficients between each scenario (**Table S2**). The average Pearson correlation coefficient across scenarios was 0.40 ± 0.14 (all individual correlations had *P* < 0.001 significance), indicating that probes tend to have varying stabilities across scenarios.

**Table 1.** Test-retest scenario descriptive statistics.

Scenario	Mean	SD	Median	Skew	Kurtosis
NoStressT1–2	0.47	0.22	0.47	0.08	−0.70
NoStressT1–3	0.46	0.22	0.45	0.12	−0.66
NoStressT1–4	0.35	0.22	0.31	0.70	−0.09
NoStressT3–4	0.49	0.25	0.50	−0.01	−0.92
NoStressT1-2-3-4	0.44	0.21	0.44	0.18	−0.42
StressT1–2	0.60	0.25	0.64	−0.47	−0.74
StressT1–3	0.60	0.24	0.64	−0.50	−0.64
StressT1–4	0.61	0.24	0.65	−0.55	−0.56
StressT3–4	0.59	0.25	0.62	−0.40	−0.76
CrossSessionT1	0.33	0.22	0.28	0.85	0.13

All descriptive statistics were calculated excluding probes with stabilities less than 0.01.

The percentages of probes in each scenario stratified by ICC value are shown in Table 2. Each scenario had a significantly different number of probes with stabilities less than 0.01 (*P* < 0.001), with NoStressT1–4 and CrossSessionT1 having the highest number of probes in this category (13.31% and 13.51%, respectively), and NoStressT1-2-3-4 having the lowest number (3.05%).

**Table 2.** Test-retest scenario probe stability distributions.

Scenario	ICC<0.01	0.01<ICC<0.50	0.50<ICC<0.75	0.75<ICC<0.90	ICC>0.90
NoStressT1–2	7.09%	51.50%	30.00%	9.00%	2.41%
NoStressT1–3	6.27%	54.44%	29.84%	7.61%	1.84%
NoStressT1–4	13.31%	66.79%	15.24%	3.34%	1.32%
NoStressT3–4	10.25%	45.41%	28.96%	11.25%	4.13%
NoStressT1-2-3-4	3.05%	59.23%	30.64%	5.18%	1.90%
StressT1–2	9.70%	30.11%	30.16%	21.51%	8.51%
StressT1–3	9.93%	28.66%	31.17%	21.51%	8.72%
StressT1–4	11.28%	27.20%	31.66%	21.82%	8.03%
StressT3–4	10.11%	31.43%	30.64%	19.93%	7.89%
CrossSessionT1	13.51%	68.05%	13.78%	3.22%	1.44%

Percentage of probes in each scenario stratified by ICC value. The total number of probes in each scenario was 843,328.

To determine the scenarios that were most closely related in terms of probe stability, hierarchical clustering was performed on the scenario ICC values. A cluster dendrogram is shown in Figure 3b displaying the relatedness of each scenario. All Stress scenarios clustered with one another while the remaining NoStress scenarios and CrossSessionT1 were clustered with one another.

### 2.3. Probe set enrichment analyses for common DNAm-based algorithms and genomic function

**Figure 4.**
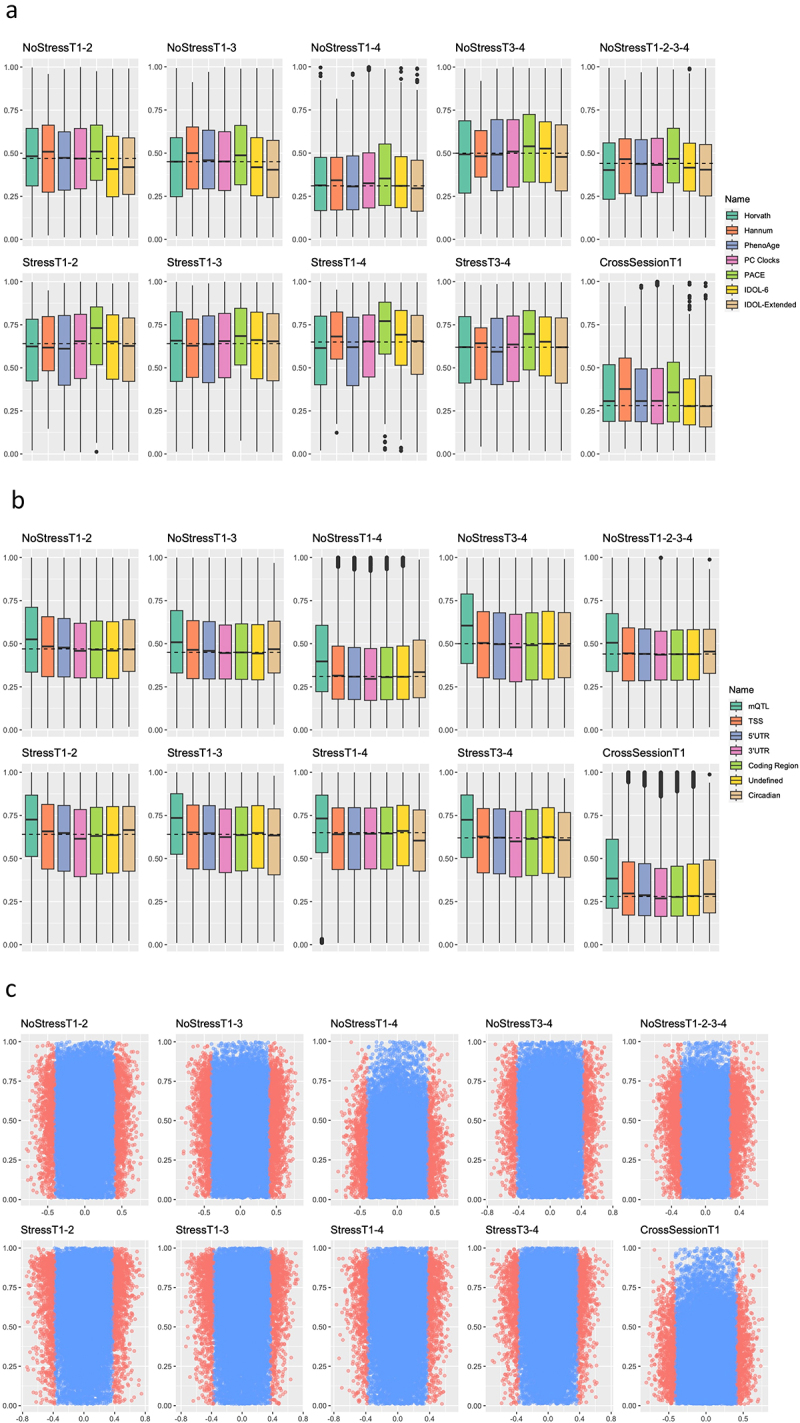
DNAm probe set enrichment analyses.

Note: All probes with ICC values less than 0.01 were excluded from analyses in this figure. The dashed horizontal lines indicate the median probe stability in each scenario (excluding probes with ICC < 0.01) and the y-axes indicate probe ICC values. A) Common DNAm clock probe set and immune cell estimator probe set distributions plotted against the median probe stability of each test-retest scenario. Horvath, Hannum, PhenoAge, PC Clock, and PACE are probes used in epigenetic clocks, while IDOL-6 and IDOL-Extended are probes used in previous and current immune cell estimators. B) Biologically relevant probe set distributions plotted against the median probe stability of each test-retest scenario. The core circadian clock genes included CLOCK, NPAS2, BMAL1, BMAL2, PER1, PER2, PER3, CRY1, and CRY2. C) Comparisons of probe stability and probe correlations with gene expression values for all test-retest scenarios. The x-axis indicates the correlation between the probe and gene expression values, and the y-axis indicates the ICC value of the probe. Blue dots are probes that were not significantly associated with gene expression values and red dots are probes that were significantly associated with gene expression values. All included probes were located in TSSs.

We tested whether sets of probes that have been used in common DNAm algorithms or that have biological significance were significantly enriched for higher ICC values in each test-retest scenario. First, we tested probe sets used in common epigenetic ageing clocks [23–27]. While Horvath, Hannum, and PhenoAge clock probe sets were not significantly enriched, Principal Component (PC) and DunedinPACE clock probes had significantly higher ICC values in most scenarios (Figure 4a and Table S3). Next, we tested whether the probe sets used in the common immune cell proportion estimators were enriched for higher ICC values. To this end, we used the EPIC IDOL-6 [28] and EPIC IDOL-Extended [29] probe libraries. Both libraries showed no significant enrichment of higher ICC values in any scenario, and the exclusion of overlapping probes between the two libraries did not change the enrichment significance results (Figure 4a and Table S3).

Probes with biological significance were also tested for their enrichment of the ICC values. Probes associated with methylation quantitative trait loci (mQTLs; see [31]) were significantly enriched in every scenario (Figure 4b and Table S3). Further, the function/genomic location of probes was also associated with whether probes were significantly more stable. Specifically, probes found in the transcription start sites (TSSs) and 5’UTRs of genes were significantly enriched in most scenarios (Figure 4b and Table S3). Conversely, probes located in 3’UTRs, coding regions, or undefined functional locations were not significantly enriched in most scenarios (**Table S3**). Finally, as expected due to diurnal changes in gene expression, probes in the TSSs of core circadian clock genes were not significantly enriched in ICC values (Figure 4b and Table S3), indicating a lack of stability in core circadian clock gene DNAm values.

Using gene expression data (see Section 4.3) in combination with the epigenetic data already detailed, we tested whether probes in gene TSSs that were significantly associated with gene expression values were enriched for higher stability in each scenario. A clear trend in enrichment scores revealed that, in all scenarios, probes significantly associated with gene expression values had significantly higher ICC values (see Figure 4c and Table S3).

### 2.4. Acute psychosocial stress significantly impacts DNA methylation around specific gene transcription start sites

To test whether any probes that were normally considered ‘highly stable’ were significantly affected by the presence of acute psychosocial stress, we generated a list composed of probes that had a stability of 0.90 or higher throughout all NoStress scenarios and CrossSessionT1 (see **Table S4** for a list of highly stable probes). We used a repeated-measures ANOVA framework to test for differences in probe values between T1 and T2, T3, or T4 in the stress session. All probes used in this analysis were located in gene TSSs, and analyses were performed using both M-values and β-values (See Section 4.2).

**Figure 5.**
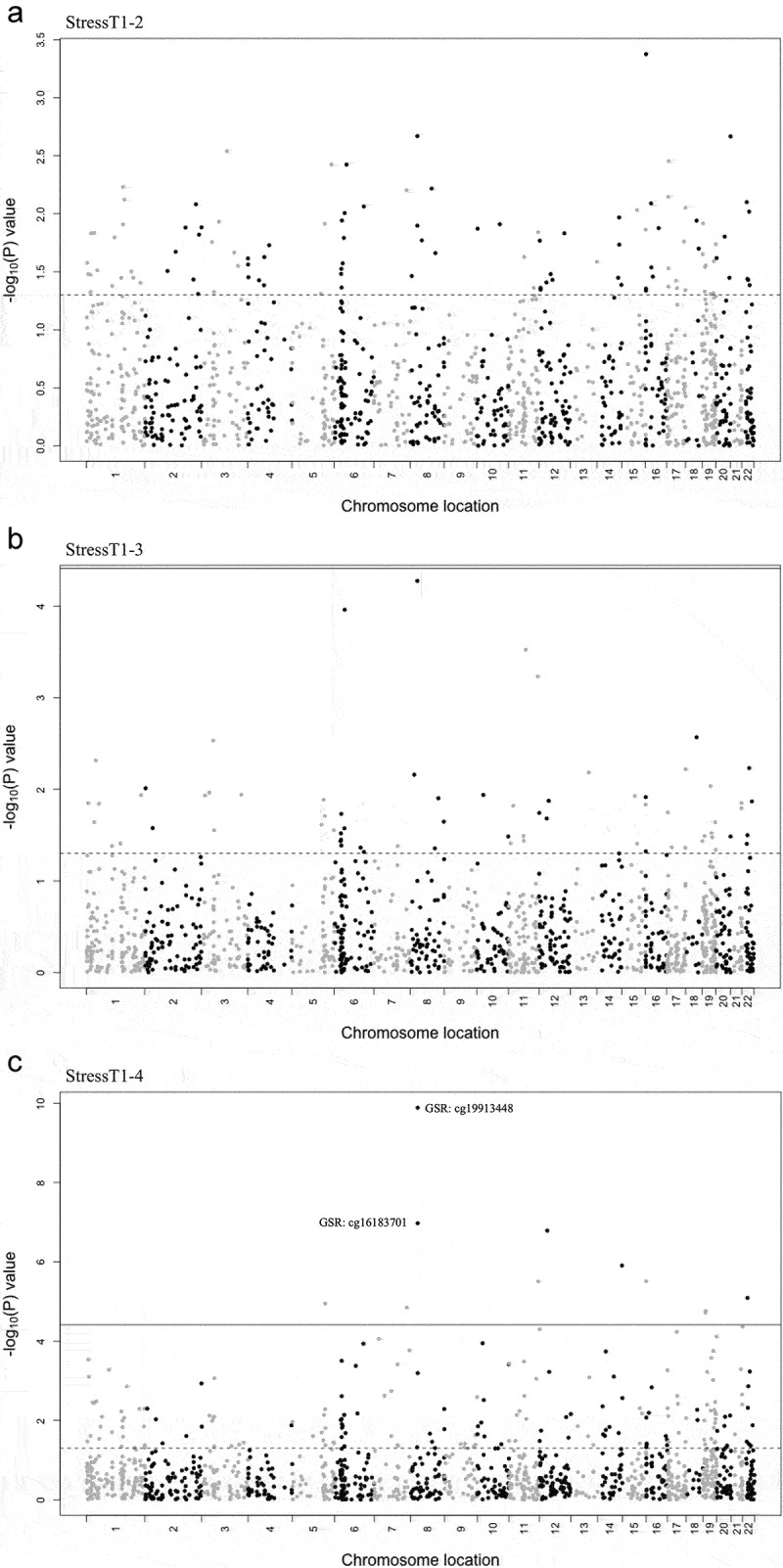
Effects of acute psychosocial stress on highly stable DNAm probes.

Note: a) Manhattan plot showing T1 – T2 stress effect significance (y-axis) and chromosomal location of the probe (x-axis). The dotted line indicates a significance threshold of 0.05. b) Manhattan plot showing T1 – T3 stress effect significance (y-axis) and chromosomal location of the probe (x-axis). The dotted line indicates a significance threshold of 0.05 and the solid line indicates a significance threshold of 3.87E-05. C) Manhattan plot showing T1 – T4 stress effect significance (y-axis) and chromosomal location of the probe (x-axis). The dotted line indicates a significance threshold of 0.05 and the solid line indicates a significance threshold of 3.87E-05. Labelled *GSR* probes cg19913448 and cg16183701 included.

No significant Bonferroni probe changes were observed between T1 and T2 during the stress session (StressT1–2; Figure 5a). Similarly, no probes were found to have significantly altered methylation levels when comparing T1 and T3 during the stress session (StressT1–3; Figure 5b). In contrast, when comparing T1 and T4 during the stress session (StressT1–4; Figure 5c), a TSS probe (cg19913448) in the glutathione-disulfide reductase (*GSR*) gene was found to have significantly reduced methylation levels (*M*=-0.191, *P* = 1.30E–10). Additionally, 10 more probes were found to be significantly demethylated when comparing T1 and T4 in the stress session (*P* < 3.87E–05; Table 3), one of which (cg16183701) was also located in the TSS of the *GSR* gene (*M*=-0.156, *P* = 1.06E–07). The results indicated significant methylation changes in highly stable probes in response to acute stress.

**Table 3.** Effects of acute stress on highly stable DNAm probes.

Probe	Gene	β-Intercept	β Time 4 Estimate	M-Intercept	M Time 4 Estimate	P-Value
cg19913448	*GSR*	0.026	−3.21E–03	−5.276	−0.191	1.30E–10
cg16183701	*GSR*	0.017	−1.66E–03	−5.908	−0.156	1.06E–07
cg13722517	*YARS2*	0.030	−3.58E–03	−5.093	−0.211	1.63E–07
cg26031613	*KLC1*	0.045	−3.77E–03	−4.972	−0.210	1.24E–06
cg25114630	*CHSY1*	0.044	−3.44E–03	−5.236	−0.147	3.09E–06
cg00635556	*EI24*	0.021	−2.08E–03	−5.611	−0.155	3.13E–06
cg13353337	*LZTR1*	0.126	−5.34E–03	−3.903	−0.190	8.14E–06
cg25613667	*WDR55*	0.164	−7.77E–03	−3.376	−0.157	1.13E–05
cg00287122	*RAB19*	0.046	−5.96E–03	−4.444	−0.224	1.43E–05
cg24400183	*ZNF563*	0.014	−2.61E–03	−6.128	−0.254	1.73E–05
cg03007623	*CDKN2D*	0.058	−2.29E–03	−5.206	−0.185	1.92E–05

All Bonferroni significant TSS probes and their associated genes obtained when comparing T4 and T1 of the stress-session. Intercepts and T4 estimates are given as both β-values and M-values. P-values are given with respect to M-values. Significance threshold: *P* < 3.87E–05.

### 2.5. Individuals having experienced early-life adversity show differential DNAm probe stability in the presence of acute psychosocial stress

**Figure 6.**
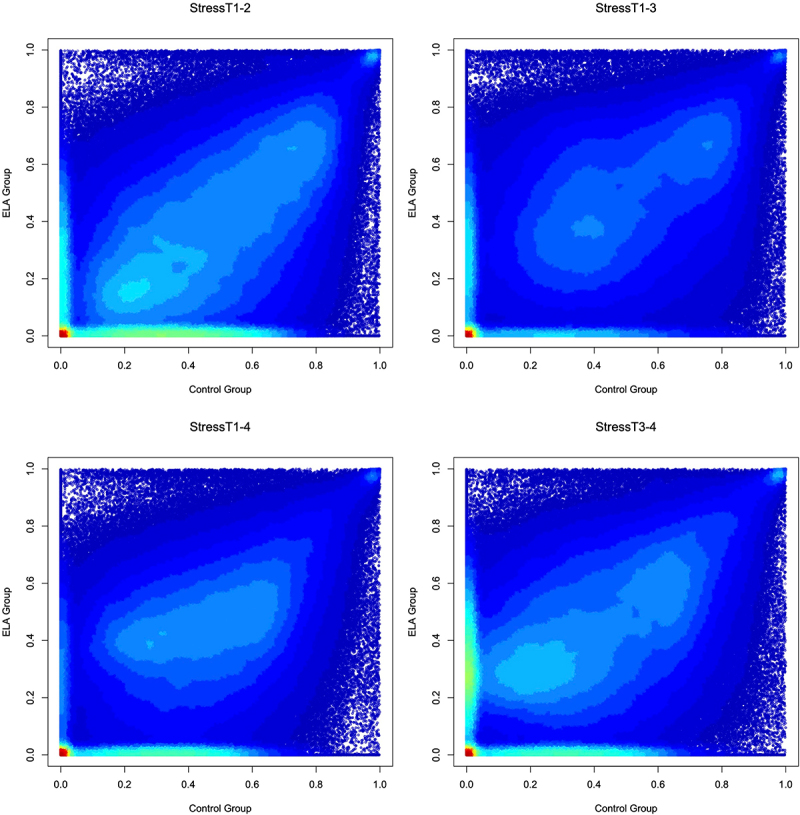
ELA vs. Non-ELA stabilities of DNAm probes in the presence of acute psychosocial stress.

Note: Heat-scatter plots are set on a three-colour gradient, with red being the densest, green being moderately dense, and blue being the least dense. White indicates no points in the region. Probe stability in the presence of acute psychosocial stress for non-ELA individuals (x-axis) and ELA individuals (y-axis) in four different scenarios.

Finally, we performed an exploratory analysis to determine whether ELA status was associated with probe stability in the presence of acute psychosocial stress. We stratified our sample by ELA status for all Stress scenarios and tested whether there was a significant mean difference in ICC values between the samples for each scenario. In all scenarios other than StressT3–4, individuals that had experienced ELA had probe stabilities that were significantly lower than probe stabilities in non-exposed individuals (StressT1–2: *β*=-0.065, *P* < 0.001; StressT1–3: *β*=-0.013, *P* < 0.001; StressT1–4: *β*=-0.024, *P* < 0.001; StressT3–4: *β* = 0.015, *P* < 0.001; Figure 6). Additionally, in all scenarios other than StressT3–4, the ELA group had a significantly higher number of probes with a low ICC < 0.01 when compared to control samples (*P* < 0.001). These exploratory analyses suggest that ELA had a significant impact on probe stability in response to stress.

## 3. Discussion

Using a unique experimental design, we tested the biological stability of EPIC array measurements under diverse conditions. We found that varying time intervals, the presence of acute psychosocial stress, and ELA exposure influenced the probe stability of repeated DNAm measurements. Additionally, we found that probes used in most epigenetic-based algorithms for estimating epigenetic age or immune cell proportions had average or below-average stability. Using stability measurements in the absence of stress, we found multiple highly stable probes in the TSS of the *GSR* gene that were hypomethylated in the presence of acute stress.

Specifically, the DNAm probes had differing stabilities when measurements were taken at varying time intervals. Interestingly, the NoStressT1–2 and NoStressT1–3 scenarios had similar mean and median probe ICC values, indicating that the probe stability remained relatively constant when comparing time intervals of 75 and 135 min. The Pearson correlation coefficient between these two scenarios also indicated that probes that had high stability over 75 min had high stability over 135 min. However, when the time interval was extended to 285 min (i.e., the NoStressT1–4 scenario), probe stability decreased, indicating that changes in probe values were more readily apparent when measurements were taken further apart. Although the experimental design included a standardized meal between T3 and T4, the meal did not account for changes in probe stability because of the high stabilities observed in the pre- and post-meal scenario (i.e., NoStressT3–4). When comparing probe measurements over one week (i.e., first measurements in each session, CrossSessionT1), the probe mean and median stabilities were lower compared to most other scenarios. However, NoStressT1–4, which spanned 285 min, had similar probe ICC values as CrossSessionT1, in which samples were obtained a week apart. The correlation between these two scenarios’ probe stabilities was 0.62, indicating that probes that were unstable over 285 min were generally unstable over one week between measurements.

Probe stability was also influenced by acute psychological stress. Comparing repeated measures in the Stress session taken over 75 and 135 min revealed similar probe stabilities, but contrary to the results from the NoStress scenarios, repeated measures spanning 285 min had similar probe stabilities to Stress scenarios spanning 75 and 135 min. The inclusion of a standardized meal in the Stress session did not affect probe stability, as evidenced by the pre- and post-meal scenario (i.e., StressT3–4) having similar probe stabilities to all other Stress scenarios. These observations suggest that acute psychosocial stress may exert a protective or stabilizing influence on DNAm patterns over multiple hours, which was not observed in the absence of a stressor. In other words, DNAm probes were *more* stable over the 285-minute period in the presence of acute psychosocial stress.

DNAm probes used in common epigenetic clocks and immune cell proportion estimators had varying distributions in each test-retest scenario. Notably, the robustness of the probes included in both the PC and DunedinPACE clocks was enriched for highly stable probes in both the absence and presence of acute psychosocial stress. In contrast, the distributions of probe stabilities used in other clocks, on average, performed at or below what we observed in the complete sample of the EPIC array probes. These findings were expected because of the use of technically reliable probes in the design of both the PC and DunedinPACE clocks. In addition, epigenetic estimators of immune cell proportions also performed, on average, at or below what we observed in the complete set of probes. This was unexpected, given the robustness of these algorithms and libraries, as reported previously [29]. These results indicate that although immune cell proportion estimators perform well, the inclusion of highly stable probes could improve their performance over a variety of temporal scenarios. Because many DNAm-based estimators include probes with low stability, we recommend using means of multiple measurements (e.g., three times over three days) to ensure a more stable estimate from these DNAm-based algorithms. Similarly, investigators conducting studies testing the effects of geroprotective interventions using epigenetic ageing as an intermediate outcome could use standardized sample collection time and multiple measurements to improve the reliability of the results.

To test whether genomic location or function was associated with probe stability, we examined the distribution of ICC values of probes in mQTLs, TSSs, 5’UTRs, 3’UTRs, coding regions, and near core circadian clock genes. Most categories of biologically relevant probes we tested performed at or below what was observed in the EPIC probe set, indicating that there is a lack of association between genomic location and probe stability. However, the probes associated with mQTLs showed significant stability enrichment in both the absence and presence of acute psychosocial stress. In essence, mQTL probes appear to be more affected by genetic variation than by time or environmental stimuli, making these probes more stable DNAm markers. In addition to the stability of mQTL-associated CpG sites, probes found in TSSs were also found to have higher stability than average in almost all scenarios, indicating that there is greater stability of probes that take place in proximal gene regulation when compared with probes located elsewhere. Finally, as expected [32], core circadian clock gene probes displayed lower than average stability, bolstering the validity of our stability estimates.

We further sought to test the reliability of probes in response to stress by extracting probes that were highly stable across both the entirety of the NoStress session and in the CrossSessionT1. In doing so, we found that there were a number of highly stable probes that were significantly altered by acute psychosocial stress, which were observed 285 min post-stressor. Notably, two probes (cg19913448 and cg16183701) near the TSS of the *GSR* gene showed significantly decreased levels of DNAm post-stressor after Bonferroni correction. *GSR* is a gene that has been implicated in the cellular detoxification response to internal and external stressors [33]. Notably, in an RNAi-knockdown experiment on *C. elegans*, *GSR* was shown to be a critical stress-response gene, with higher levels of *GSR* providing a protective effect against stress (sub-lethal concentrations of juglone, a toxic chemical to *C. elegans*) and increasing longevity [34]. When examining gene expression data for *GSR*, we did not see any significant expression differences in response to acute psychosocial stress. Therefore, although *GSR* expression did not change in response to stress in our study, nor did it differ by ELA status, it is possible that alterations in the DNAm patterns of the *GSR* TSS may be a mechanism of adaptation to future stress. Alternatively, it is possible that we did not measure gene expression over a sufficiently-long time interval to detect changes in *GSR* expression.

Individuals with ELA exhibited greater DNAm instability in the presence of acute psychosocial stress than non-ELA-exposed individuals. The greatest difference in probe stability between the ELA and control groups was observed 30 min post-stressor, suggesting that the global DNAm profile of individuals with ELA was altered in the presence of acute psychosocial stress over short time intervals. Differences in global probe stabilities of ELA and non-ELA individuals decreased as time passed since the stressor. Although DNAm has previously been viewed as a more static molecular marker of cellular identity, some research suggests that methylation levels of multiple probes are subject to temporal instability [13,35]. These previous findings, along with our results, suggest that DNAm instability may be a possible mechanism for differences in physiological and molecular responses to stress in individuals with ELA.

We acknowledge limitations of this study. First, we attempted to account for PBMC subtype proportions by controlling for monocytes rather than additionally controlling for lymphocyte subtypes (i.e., CD4 memory, CD4 naïve, T cells, NK cells, etc.). Although this simplified our analyses, this choice did not allow us to consider the varying proportions of lymphocyte subsets; therefore, we did not consider the differing methylation profiles of lymphocyte subsets. Second, although we assessed multiple repeated measures over varying intervals of time and our experimental design included both within-person and between-group measurements, we had a relatively small sample size for most scenarios. Because we observed changes in the distributions of probe stability when increasing our sample size, we recommend that our results be taken with caution until further analyses with larger sample sizes are performed. Finally, as we performed these analyses in young adults, our results should not be considered representative of the general population without additional analyses performed in larger and more diverse samples.

Contrary to the common view that DNAm is a stable marker of cellular identity, we present evidence that DNAm is a dynamic molecular marker. Owing to the dynamic nature of DNAm, investigators implementing study designs using epigenetic-based estimators in the presence of stress should be aware that the probes used in some algorithms (i.e., the PC and DunedinPACE clocks) are more robust to DNAm instability than others. Future DNAm-based algorithms should incorporate highly stable probes that are unaffected by either temporal proximity or acute psychosocial stress. In addition, we have shown that normally highly stable probes in the TSS of the *GSR* gene decrease stability in the presence of acute psychosocial stress, and that individuals who have experienced ELA have less stable global DNAm profiles in the presence of acute psychosocial stress. Future work should investigate DNAm alterations in specific immune cell subtypes over time (e.g., monocytes, neutrophils, B cells, T cells, etc.), both in the absence and presence of acute psychosocial stress and in ELA- and non-ELA-exposed populations. Overall, our results indicate that both the presence of acute psychosocial stress and prior adverse experiences can influence probe stability, and that DNAm profiles are dynamic across time and in the presence of acute psychosocial stress.

## 4. Materials and methods

### 4.1. Study participants

This study included a sample of 34 healthy individuals aged 18–25 years. Biological samples were collected from participants at 8 separate time points. Among all samples, 10 biological samples did not pass DNAm average detection p-value filtering (see *4.2 – DNA Methylation*), resulting in a maximum number of individuals in each scenario of 31. Participants were recruited by word-of-mouth and advertisements on campus bulletin boards at The Pennsylvania State University. During a visit to The Pennsylvania State University’s Clinical Research Centre, participants were subjected to the Trier Social Stress Test (TSST), a laboratory psychosocial stress protocol shown to induce robust hypothalamic-pituitary-adrenal axis and sympathetic nervous system activation [36], followed by a 4-hour post-test sampling and questionnaire period. Additionally, each participant attended a no-stress control visit one week apart, with randomization for the visit order among participants. In both sessions, testing began at 11:00 am and ended at 4:15pm. Blood was drawn at four points during these sessions (30 min before the TSST and 30, 90, and 240 min post-TSST), and a standardized meal was given to each participant between the third and fourth blood draws at each session (Figure 1). Participants were given specific instructions to refrain from excessive physical activity on the days of the testing, consuming alcohol for 12 h before their arrival, and eating and drinking (besides water) for 2 h prior to the testing sessions. The TSST was scheduled to begin at 12:00 pm to minimize the effects of circadian changes in cortisol, and lasted for approximately 15 min. Detailed information on the study procedures, including details of the TSST, has been reported previously [37].

The original motivation for this data collection was to examine the differences in gene expression due to ELA status. For the purpose of the current investigation, we combined data from all participants, including those who had experienced ELA and controls. Additional analyses further examined the differences in probe reliabilities by ELA status. The ELA status of participants was assessed by a trained clinical interviewer during a phone interview using the Stressful Life Events Screening Questionnaire, as described previously [37]. No significant demographic differences (sex, age and minority status) between ELA and control groups were observed (*P* > 0.05; see **Table S5**).

### 4.2. DNA methylation

Whole blood samples were repeatedly collected via an intravenous catheter into the antecubital vein, resulting in 192 samples. Blood samples were collected in 10 mL EDTA tubes and immediately centrifuged for 10 min at 1500 g prior to collection of plasma. PBMCs were isolated via density-gradient centrifugation using Ficoll. A small fraction of granulocytes in PBMC samples may have been retained (mean remaining granulocyte composition was 1.5% ± 5.4%, according to DNAm estimates) during processing, which was similar across repeated samples.

DNA was extracted from PBMCs using the QIAamp mini kit (Qiagen) and sent to the Genome Sciences Core at The Pennsylvania State University for whole genome DNAm analysis using the Infinium MethylationEPIC array (EPIC; Illumina, CA, USA). The eight biological replicates for each individual were placed on a single slide, such that each individual received their own slide (8 wells). We used two arrays (12 slides per array) for all samples and all ICC value calculations and analyses run were performed controlling for array number (see *4.5 – ICC Value (Stability) Calculations*). We refer to the process of controlling for array number as controlling for ‘batch effects.’ EPIC array IDAT imaging files were converted to DNAm *β* value matrices with the minfi [38] package using R statistical software (R v4.1.2). Because of heteroscedasticity, *M* value matrices were also created for individual statistical testing, as suggested previously [39] (see Section 2.4; Table 3). All but ten samples passed the average probe detection p-value filtering (*P* < 0.05), which resulted in a total sample size of 31 individuals and 182 individual measurements. Subsequently, probes with a detection p-value greater than 0.05 in more than 10% of samples were excluded, along with probes on sex chromosomes, which resulted in a total of 843,328 remaining probes. Cross-hybridizing probes, probes not at CpG sites, probes with high bead counts and probes overlapping with SNPs were not excluded in order to mirror the analyses performed by Sugden et al. [6]. Additionally, although these types of probes were included, all probes with ICC < 0.01 were excluded from the majority of analyses in the present study. Sample normalization was performed using the noob normalization method in the minfi package [38].

### 4.3. Gene expression measurements and preprocessing

Gene expression data were collected from RNA extracted from PBMCs. Samples were delivered to the Genome Sciences Core at The Pennsylvania State University, where RNA quality was verified using the Agilent Bioanalyzer, followed by library construction. Library preparation was performed using the QuantSeq 3’mRNA-Seq Library Prep Kit FWD for Illumina (Lexogen) supplemented with UMI (unique molecular index) according to the manufacturer’s instructions. Sequencing was performed using an Illumina Nova-seq 6000. BBDuk (sourceforge.net/projects/bbmap/) was used for quality control, with a minimum read length of 25bp. Quality trim for each group of reads was set to Q10 using the Phred algorithm. A conserved sequence of 12bps was trimmed from the start of each read, and all reads were trimmed to end at bp 70. All sequencing run samples were decontaminated using BBDuk’s standard contamination library. Reads were then aligned to the human reference genome (GRCh38_latest_genomic.fna) using STAR [40]. Reads were counted with featureCounts [41] using human reference genome annotation (GRCh38_latest_genomic.gff). ‘ID’ and ‘gene’ were the feature specifications used for – g and – t flags of featureCounts, respectively. Transcript counts were converted to counts per million (CPM), filtered to include only transcripts that had a CPM value greater than 1 for 90% or more of the sample, normalized using the TMM method, and subsequently log2 transformed.

### 4.4. Enrichment analysis probe sets and estimation of immune cell subtype proportions

Probes used in epigenetic-based algorithms and those with biological significance were used for enrichment analyses. The epigenetic ageing clocks used were the Horvath multi-tissue clock [23] (*n* = 353), Hannum blood clock [24] (*n* = 89), Levine PhenoAge clock [25] (*n* = 513), PC clock [27] (*n* = 78,464), and DunedinPACE clock [26] (*n* = 173). The immune cell proportion estimators used were EPIC IDOL-6 [29] (*n* = 450) and EPIC IDOL-Extended [28] (*n* = 1,200). Due to an abundance of previous work investigating the reliability of epigenetic algorithms [23–29], only individual probes from these epigenetic algorithms were tested for stability. mQTL probes were obtained from previous research [31] and TSS, 5’UTR, 3’UTR, coding region, and undefined probes were obtained from the *IlluminaHumanMethylationEPICanno.ilm10b4.hg19* package in R, which provides annotations of genomic location for each EPIC array probe. The core circadian clock genes included were CLOCK, NPAS2, BMAL1, BMAL2, PER1, PER2, PER3, CRY1, and CRY2.

Estimates of immune cell subtype proportions were computed from the DNAm data using the ProjectCellType_CP function in the FlowSorted.Blood.EPIC package, which is equivalent to the ProjectCellType function in minfi. The ‘FlowSorted.BloodExtended.EPIC’ library [29] was used as reference data for blood cell proportion estimates.

### 4.5. ICC value (stability) calculations

The stability of the probes across different time points was quantified using ICC estimates. The ICC values for each scenario were calculated using the normalized beta values for each probe present on the Illumina EPIC methylation array. Because there are multiple methods of calculating ICC values [30], two types of ICC values were computed: two-way random effects, absolute agreement, single rater model (2,1), and random effects, absolute agreement, and multiple rater model (2,k). As probe DNAm values were measured using PBMCs, which consist primarily of monocytes and lymphocytes, and cell proportions changed over time [42], ICC values were adjusted by the estimated monocyte proportions. Adjustment was achieved by adding monocyte percentage as a covariate in the linear mixed-effects models. The model equations and ICC calculation formulae are as follows:

Unadjusted model and ICC values:

$$y_{it}=\beta_{0}+\omega_{i}+\nu_{t}+\varepsilon_{it}$$

$$ICC{\left(2,1\right)}_{=}\frac{\sigma_{\omega }^{2}}{\sigma_{\omega }^{2}+\sigma_{\nu }^{2}+\sigma_{\varepsilon }^{2}}$$

$$ICC\left(2,k\right)=\frac{n_{t}\sigma_{\omega }^{2}}{n_{t}\sigma_{\omega }^{2}+\sigma_{\nu }^{2}+\sigma_{\varepsilon }^{2}}$$

Adjusted model and ICC values:

$$y_{it}=\beta_{0}+\beta_{1}x_{1it}+\beta_{2}x_{2it}+\omega_{i}+\nu_{t}+\varepsilon_{it}$$

$$ICC{\left(2,1\right)}_{adj}=\frac{\sigma_{\omega }^{2}}{\sigma_{\omega }^{2}+\sigma_{\nu }^{2}+\sigma_{\varepsilon }^{2}}$$

$$ICC{\left(2,k\right)}_{adj}=\frac{n_{t}\sigma_{\omega }^{2}}{n_{t}\sigma_{\omega }^{2}+\sigma_{\nu }^{2}+\sigma_{\varepsilon }^{2}}$$

In the equations above, $y_{it}$ is the DNAm probe value of the *i*th individual at time point t, $\beta_{0}$ is the grand intercept, $\beta_{1}$ is the regression coefficient for monocyte percentage, $x_{1it}$ is the monocyte percentage of the *i*th individual at time point t, $\beta_{2}$ is the regression coefficient for batch effects, $x_{2it}$ is the array number of the *i*th individual at time point t, $\omega_{i}$ is the individual-level random effect with a mean of 0 and variance of $\sigma_{\omega }^{2}$, $\nu_{t}$ is the time-point-level random effect with a mean of 0 and variance of $\sigma_{v}^{2}$, $\varepsilon_{it}$ is the residual with a mean of 0 and variance of $\sigma_{\varepsilon }^{2}$, $n_{t}$ is the number of time points included in the analysis, and $ICC{\left(2,1\right)}_{adj}$ and $ICC{\left(2,k\right)}_{adj}$ are the adjusted ICC(2,1) and ICC(2,k) values, respectively.

### 4.6. Description of test-retest scenarios

We calculated the stability of the DNAm probes across a variety of scenarios (Figure 1). The names of each scenario are shown on the left-hand side of Figure 1. All scenarios included two repeated measurements of the DNAm probe values from the same individual, with the exception of NoStressT1-2-3-4, which included four repeated measurements from the same individual. Scenarios were designed to test the stability of the DNAm probes over varying lengths of time in both the absence and presence of acute psychosocial stress. A detailed description of each scenario is presented in **Table S1** and in the *Introduction*.

### 4.7. Analysis plan

We tested whether probe stability was dependent on the type of ICC value used, immune cell proportions, sample size, and number of repeated probe measurements. To test whether these factors were significantly associated with probe stability, we took advantage of different combinations of test-retest scenarios. We used paired t-tests to compute differences in average probe reliabilities and Chi-square tests to determine if there were differences in the counts of probes with ICC < 0.01 and probes with ICC ≥ 0.01. Heat-scatter plots were generated for each of the study design variables tested, and each scenario was compared.

We generated histograms of probe stability for each test-retest scenario (excluding probes with ICC < 0.01). Hierarchical clustering for different scenarios was performed on scenario ICC values for each probe using the *hclust* function in the R statistical software (R v4.1.2). As with the generation of histograms, hierarchical clustering was performed excluding probes with an ICC < 0.01.

We determined the correlation between methylation status and gene expression values of probes in TSSs using the *IlluminaHumanMethylationEPICanno.ilm10b4.hg19* library in R to extract probes in TSSs. Gene expression values were correlated with DNAm probes by computing the Pearson correlation coefficients of the corresponding DNAm probes and genes. Enrichment analyses of probe stability were performed using the *fgsea* [43] package in R, where 10,000 permutations were performed, and all probe sets were limited to 35,000 probes (randomly sampled from a larger probe set if too large). All enrichment analyses (probes used for epigenetic clocks, immune cell estimators, and biologically relevant probes) were performed in the same manner.

We created a list of highly stable probes by extracting probes with ICC > 0.90 in all NoStress scenarios and in the CrossSession scenario. Differences in highly stable probe DNAm values in response to acute psychosocial stress were computed using a repeated-measures ANOVA framework, controlling for immune cell proportions and DNAm batch effects.

The effects of ELA on probe stability in the presence of acute psychosocial stress were tested using paired t-tests and Chi-square tests, as described above.

## Supplementary Material

Supplemental Material

## Data Availability

The code used to perform all the analyses is available at: https://github.com/abnerapsley1/DNAmStabilityMeasurements. Data used in this publication have been deposited in NCBI’s Gene Expression Omnibus (GEO) and are accessible through the GEO Series accession number GSE227815 (https://www.ncbi.nlm.nih.gov/geo/query/acc.cgi?acc=GSE227815).
